# Developmental Neurotoxicity of Difenoconazole in Zebrafish Embryos

**DOI:** 10.3390/toxics11040353

**Published:** 2023-04-08

**Authors:** Qing Yang, Ping Deng, Dan Xing, Haoling Liu, Fang Shi, Lian Hu, Xi Zou, Hongyan Nie, Junli Zuo, Zimeng Zhuang, Meiqi Pan, Juan Chen, Guangyu Li

**Affiliations:** 1 Institute of Hydroecology, Ministry of Water Resources & Chinese Academy of Sciences, Wuhan 430079, China; 2 Wuhan Academy of Agricultural Sciences, Wuhan 430072, China; 3 Dadu River Hydropower Development Co., Ltd., Chengdu 610016, China; 4 College of Fisheries, Huazhong Agricultural University, Wuhan 430070, China; 5 Changsha Xinjia Bio-Engineering Co., Ltd., Changsha 410000, China

**Keywords:** difenoconazole, zebrafish, developmental neurotoxicity, neurotransmitter content, AChE activity

## Abstract

Difenoconazole is a type of triazole fungicide that is widely used in the treatment of plant diseases. Triazole fungicides have been shown in several studies to impair the development of the nervous system in zebrafish embryos. There is still little known about difenoconazole-induced neurotoxicity in fish. In this study, zebrafish embryos were exposed to 0.25, 0.5, and 1 mg/L of difenoconazole solution until 120 h post-fertilization (hpf). The difenoconazole-exposed groups showed concentration-dependent inhibitory tendencies in heart rate and body length. Malformation rate and spontaneous movement of zebrafish embryos increased, and the locomotor activity decreased in the highest exposure group. The content of dopamine and acetylcholine was reduced significantly in difenoconazole treatment groups. The activity of acetylcholinesterase (AChE) was also increased after treatment with difenoconazole. Furthermore, the expression of genes involved in neurodevelopment was remarkably altered, which corresponded with the alterations of neurotransmitter content and AChE activity. These results indicated that difenoconazole might affect the development of the nervous system through influencing neurotransmitter levels, enzyme activity, and the expression of neural-related genes, ultimately leading to abnormal locomotor activity in the early stages of zebrafish.

## 1. Introduction

In recent years, the total global population has been growing, with the development of international economic integration and the improvement of standards of living putting forward higher requirements for the yield and quality of crops. As the demand for agricultural products expands, crop resistance to pests and diseases increases considerably, which gradually leads to the increase in the use of pesticides. Triazole fungicides are widely used in agriculture production to protect a wide range of crops due to their broad spectrum, high effectiveness, and long-duration effects [1,2]. One of the most commonly used triazole fungicides for preventing and treating diseases is difenoconazole. By blocking lanosterol-14-α-demethylase (Cyp51) activity, difenoconazole can interfere with the structure and function of cell membranes, ultimately inhibiting fungal growth [3,4,5]. Therefore, difenoconazole is widely used throughout the world, and the demand in the global market is gradually increasing.

Difenoconazole could penetrate nearby soil and water environment during application [6]. Because of its properties of long-term retention, limited biodegradability, and ease of movement in the environment, difenoconazole could remain in the environment for a long time and exert harmful effects on soil and aquatic creatures [7]. In recent years, the concentration of difenoconazole in aquatic environments has been frequently detected around the world. For example, it was detected in a rice field up to 20 days after irrigation in Brazil, with concentrations ranging from 0.4 µg/L to 36.2 µg/L [8]. In the drainage around paddy fields in Malaysia, the concentration of difenoconazole was detected to be 300 μg/L after 7 days of irrigation [9]. In China, the concentration of difenoconazole in paddy water was found to be between 1.98 and 2.91 mg/L [10]. With the exception of paddy water, difenoconazole was found in Australian surface water at a concentration of 0.15 µg/L [11]. With respect to its widespread application for improving crop yields, the risk of difenoconazole to aquatic creatures should not be ignored.

Previous studies have shown difenoconazole may have multiple deleterious impacts on organisms throughout their life cycle [12]. Fish are in direct contact with contaminated water and can be utilized to assess the ecotoxicological effects of difenoconazole. Jiang et al. [13] found that difenoconazole can cause hepatotoxicity as well as interfere with lipid metabolism and the balance of intestinal microbiota. Long-term exposure to difenoconazole causes accumulation in marine medaka and zebrafish while reducing next-generation viability [14,15]. Mu et al. [16] reported that zebrafish embryos displayed hatch inhibition, decreased heart rate, and abnormal swimming behavior after difenoconazole exposure. Further study has confirmed that difenoconazole could impair the heart development of zebrafish via oxidative stress-mediated apoptosis pathway [17]. However, few studies on the neurodevelopmental toxicity of difenoconazole in zebrafish in their early life stages have been published.

Behavior is a result of the activity of the nervous system, which is necessary for fish to survive and reproduce in the wild [18]. When fish are confronted with environmental stressors, their behavior will respond accordingly. The generation of fish behavior involves a variety of physiological and biochemical processes, including the transmission of chemical signals [19]. There is increasing evidence that potential neurotoxic compounds (such as pesticides and heavy metals) and drugs (such as tranquilizers) can affect the development of the nervous system by interfering with the dopamine (DA) nervous system and cholinergic nervous system, resulting in behavioral changes in zebrafish larvae [20,21,22]. Previous research has revealed that exposure to neurotoxins during critical stages of development can cause damage to the nervous system of the adult brain [23,24]. Thus, it is imperative to explore the effects of difenoconazole on neurodevelopment of zebrafish embryos.

Zebrafish embryos are an ideal model for assessing the neurodevelopmental toxicity of difenoconazole due to their transparency, the fact that they develop rapidly, their fully sequenced genome, and their similarity to the mammalian neurotransmitter system [25]. In the present study, after exposure to different concentrations of difenoconazole solution for 5 days, its effects on zebrafish developmental endpoints, motor behavior, neurotransmitters content, acetylcholinesterase (AChE) activity, and related gene expression were evaluated, with the purpose of probing into the damaging effect of difenoconazole on the zebrafish nervous system and its possible mechanism. The findings of this study will be valuable in establishing appropriate preventative and control measures, as well as providing an ecological risk assessment of difenoconazole.

## 2. Materials and Methods

### 2.1. Reagents

Difenoconazole (CAS Number 119446-68-3; purity ≥ 95%) was purchased from Sigma-Aldrich (St. Louis, MO, USA). The stock solution was prepared with 0.01% (*v*/*v*) dimethyl sulfoxide (DMSO) and stored in dark at 4 °C. All the other chemicals used in this study were analytical grade.

### 2.2. Test Organisms

Zebrafish were acquired from the Institute of Hydrobiology, Chinese Academy of Sciences (Wuhan, China) and maintained as previously described [26]. Adult zebrafish were maintained at 28 ± 1 °C with a light–dark cycle of 14 h/10 h under a closed flow-through system. Fish were fed twice a day with brine shrimp. Fish in groups of 3 males and 3 females were placed in a tank overnight to produce fertilized eggs. Normally developed embryos were arbitrarily sorted and incubated in embryo medium (60 µg/mL of sea salts in distilled water), then exposed to various difenoconazole concentrations (0, 0.25, 0.5, and 1 mg/L) within 2 h post-fertilization (hpf). The exposure concentration ranges were in accordance with previous studies [27,28]. Each difenoconazole-treated group had three replications, and each of them contained 150 embryos. To ensure the solution environment was clean, the exposure solution was changed and dead embryos or larvae were removed every day until 120 hpf. The endpoints including hatching rate, mortality rate, malformation rate, heart rate, and body length were evaluated as in the previous study [29]. There were at least three duplicates of each concentration. The DMSO-treated group was utilized as a control group to assess all endpoints in this study, since preceding studies have demonstrated that 0.01% (*v*/*v*) DMSO solvent control had negligible impact on zebrafish growth and behavior [30,31]. All zebrafish experiment procedures were in compliance with the Institutional Animal Care and Use Committee of Huazhong Agricultural University.

### 2.3. Locomotor Behavior Assay

The spontaneous movement test was recorded at 24 hpf via a CCD camera stereomicroscope (Leica M205FA, Germany) for 1 min, as described in the previous study [32]. All embryos were acclimated to the environment for 5 min before video recording. A total of 30 embryos from 3 replicates were used to evaluate the frequency of embryonic spontaneous movements.

At 120 hpf, the locomotor activity assessment was carried out with a Danio Vision™ behavior instrument (Noldus B.V., Wageningen, the Netherlands) following the protocol described by Fan et al. [33]. In total, 24 larvae of the same concentration were placed in a 24-well plate (one larva per well), and each well received 1 mL of the corresponding exposure solution. After acclimation of 10 min in the behavior observation chamber, the locomotion activity analysis was conducted under a continuous visible light and subsequent dark period of photoperiod stimulation (5 min light–5 min dark–5 min light–5 min dark) at a constant temperature of 28 °C. The data were further analyzed using EthoVision^®^ XT 14 video tracking software.

### 2.4. Enzymatic Activity and Neurotransmitter Assay

Approximately 100 zebrafish larvae from each Petri dish were taken as one sample and pooled and dried in centrifuge tubes for weighing. Each sample was homogenized with Phosphate Buffer Saline (PBS, 1%, pH 7.4) (1:9, *w/v*) under ice-cold conditions. The supernatant was collected for assaying the content of specific substances and enzyme activity after centrifugation at 3500 rpm for 15 min at 4 °C. The commercial kits for determining protein concentration, acetylcholine (ACh) and DA ELISA were purchased from Nanjing Jiancheng Bioengineering Institute (Nanjing, China), while the AChE activities kit was obtained from KeyGen Biotech (Nanjing, China). The detection limit of DA was 0.05–20 ng/mL. Measurements were carried out according to manufacturer’s protocol.

### 2.5. Gene Expression Analysis

After exposing for 120 h, 30 larvae were arbitrarily collected from each parallel plate and stored at −80 °C until further analysis. The total RNA was extracted from whole larvae using Trizol Reagent (TAKARA Biotechnology, Kusatsu, Japan). Based on the OD260/OD280 ratio, the concentration and quality of RNA in the range of 1.8–2.0 were used for subsequent experiments. The cDNA synthesis, real-time PCR, and other subsequent operations were carried out in light of our previous study [28]. Sequences of primers of the related genes were designed by the Primer 3 software (http://frodo.wi.mit.edu/, accessed on 27 December 2021) or obtained from the previous study [28], and the primer sequences are provided in Table 1. Quantitative real-time polymerase chain reaction (qRT-PCR) was conducted by an ABI 7300 system (Applied Biosystem, Foster City, CA, USA) using SYBR Green PCR kits (Takara, Dalian, China). The β-actin gene was chosen as an internal standard, while the relative expression levels of the genes were calculated by the 2^−ΔΔCt^ method. The heat map was drawn using GraphPad Prime 9.0 software.

### 2.6. Statistical Analyses

The Kolmogorov–Smirnov test and Levene test were conducted using SPSS 25.0 (SPSS, Chicago, IL, USA) to verify the normal distribution and homogeneity of variance. One-way analysis of variance (ANOVA) with Tukey’s multiple comparison method was used to assess the significant differences between the control and exposure groups. The data expressed as the mean ± standard error (SEM), with a value of *p* < 0.05, were considered statistically significant.

## 3. Results

### 3.1. Developmental Toxicity

**Figure 1:**
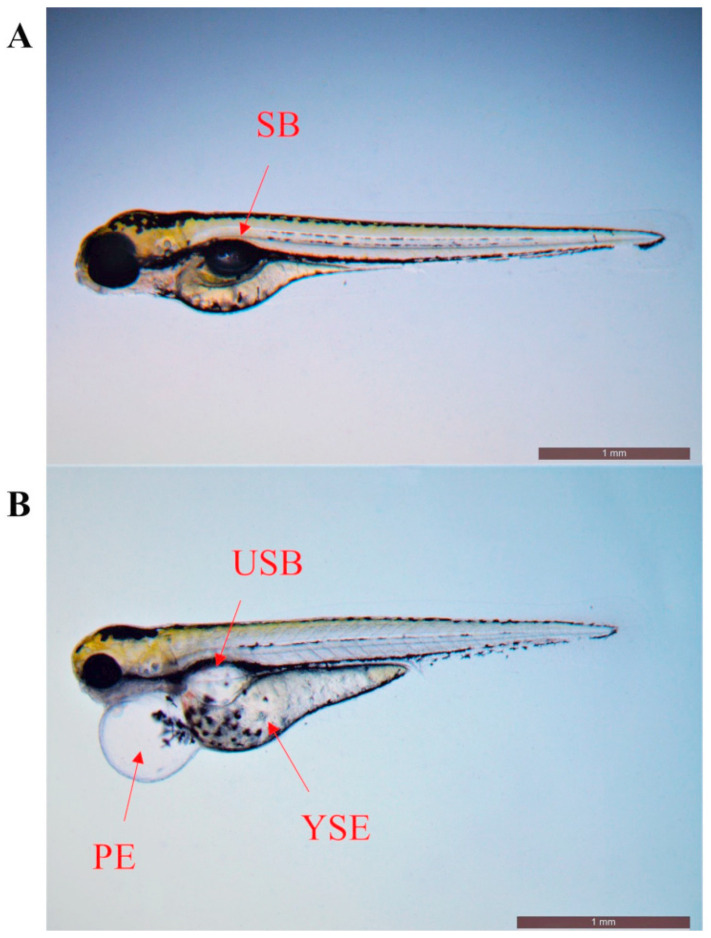
Images of zebrafish larvae at 120 hpf in control (**A**) and 1 mg/L difenoconazole exposure (**B**) groups. SB: swim bladder; USB: uninflated swim bladder; PE: pericardial edema; YSE: yolk sac edema.

To investigate the effects of difenoconazole on zebrafish embryonic development, the survival rate, hatching rate, malformation rate, heart rate, and body length were measured (Table 2). The results indicated that exposure to difenoconazole (0, 0.25, 0.5, and 1 mg/L) did not significantly affect survival rate and hatching rate of the zebrafish larvae. However, the malformation rate was significantly altered in the highest difenoconazole exposure group (*p* < 0.05). Compared to the control group, difenoconazole could cause developmental deformities of zebrafish larvae, including pericardial edema, yolk sac edema, and uninflated swim bladder (Figure 1). The heart rate and body length were reduced in the 1 mg/L difenoconazole treatment group at 120 hpf (*p* < 0.05).

### 3.2. Alterations in Behavior

**Figure 2:**
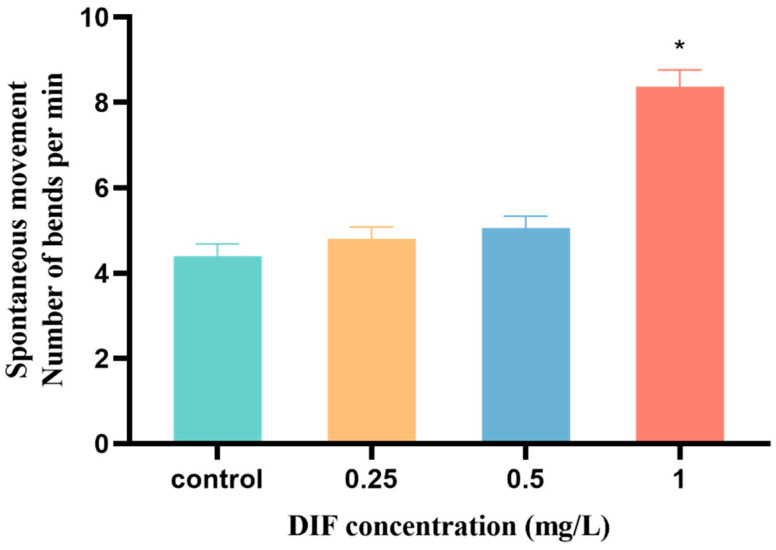
Spontaneous movement of zebrafish embryos exposed to difenoconazole (0, 0.25, 0.5, and 1 mg/L) at 24 hpf. Values are presented as the mean ± SEM with 3 replicates (10 larvae/replicate). * *p* < 0.05 indicates significant differences between exposure groups and the control group.

As shown in Figure 2, the spontaneous movement was 4.40 ± 0.28 bents/min in the control group at 24 hpf. In the difenoconazole exposure groups (0.25, 0.5, and 1 mg/L), spontaneous movement occurred 80 ± 0.28 bents/min, 5.07 ± 0.27 bents/min, and 8.37 ± 0.39 bents/min, respectively. No significant alterations of spontaneous movement frequency were observed in the 0.25 and 0.5 mg/L groups. Compared to the control group, the spontaneous movement of larvae in the 1 mg/L difenoconazole exposure group was considerably enhanced by 90.23% (*p* < 0.05).

**Figure 3:**
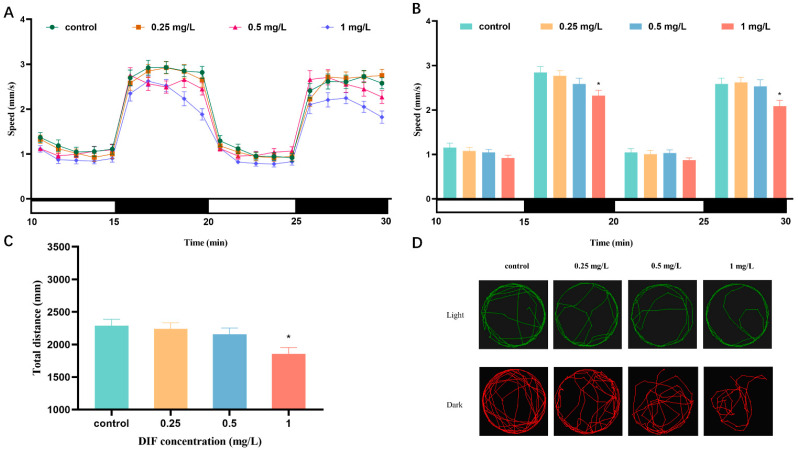
The locomotor behavior of zebrafish larvae after difenoconazole exposure (0, 0.25, 0.5, and 1 mg/L) under light–dark photoperiod stimulation test at 120 hpf. (**A**) Locomotor patterns in response to light–dark change, (**B**) the average swimming speed, (**C**) the total distance of zebrafish larvae under light-dark cycle, and (**D**) representative locomotion tracking under light and dark conditions. Values are expressed as mean ± SEM of 3 replicates (8 larvae/replicate). * *p* < 0.05 indicates significant differences between exposure groups and the control group.

The locomotor activities were assessed at 120 hpf (Figure 3). During the light stage, there was no significant difference in average speed between the difenoconazole-treated groups and the control group; however, the average speed significantly reduced (23.2%, *p* < 0.05) in the 1 mg/L difenoconazole treatment group during the dark stage (Figure 3A,B). Treatments with difenoconazole at 1 mg/L significantly reduced (18.6%, *p* < 0.05) the total distance zebrafish larvae traveled (Figure 3C). The locomotion tracking under dark circumstances (Figure 3D) demonstrated the highest concentration of difenoconazole resulted in decreased swimming activity of zebrafish larvae.

### 3.3. Neurotransmitter Content

**Figure 4:**
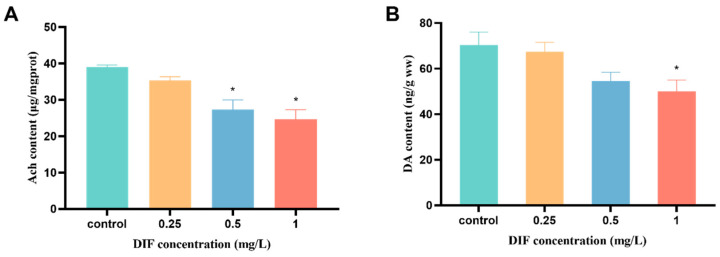
Concentrations of ACh (**A**) and DA (**B**) measured in zebrafish embryos exposed to difenoconazole (0, 0.25, 0.5, and 1 mg/L) until 120 h. Values are expressed as mean ± SEM of 3 replicates (200 larvae/replicate). * *p* < 0.05 indicates significant differences between exposure groups and the control group.

The changes in neurotransmitter content in larvae at 120 hpf are presented in Figure 4. The ACh content in 0.5 and 1 mg/L difenoconazole exposure groups were significantly decreased 29.9% and 36.7%, respectively (*p* < 0.05, Figure 4A). In addition, a dose-dependent reduction in DA levels was also observed in zebrafish larvae, with a significant decrease by 29.0% in the 1 mg/L difenoconazole-exposed group (*p* < 0.05, Figure 4B).

### 3.4. AChE Activity

**Figure 5:**
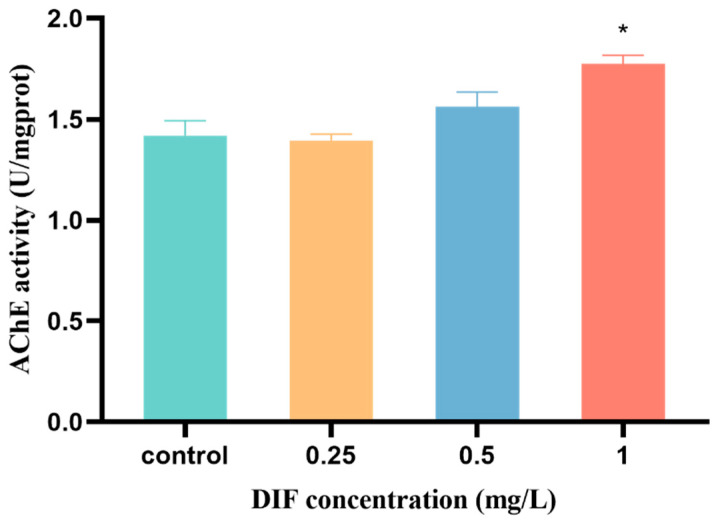
The AChE activity in zebrafish embryos measured following exposure to difenoconazole (0, 0.25, 0.5, and 1 mg/L). Values are expressed as mean ± SEM of 3 replicates (100 larvae/replicate). * *p* < 0.05 indicates significant differences between exposure groups and the control group.

Compared with the control group, the activity of AChE was markedly induced (by 25.0%) at the highest difenoconazole exposure group, whereas the activity had no marked changes in the 0.25 mg/L and 0.5 mg/L difenoconazole exposure groups (*p* < 0.05, Figure 5).

### 3.5. Gene Expression

**Figure 6:**
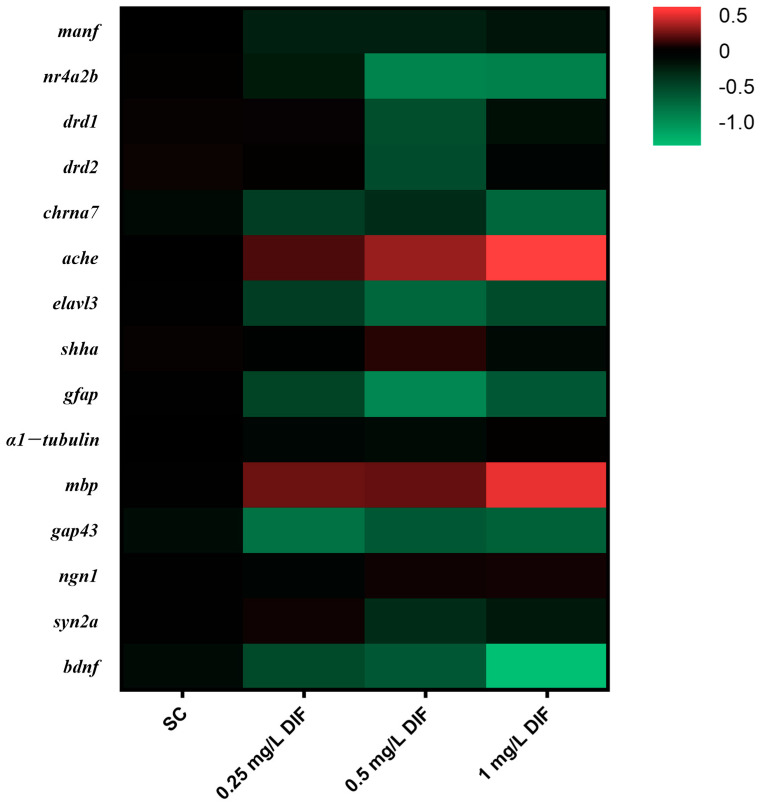
Heat map of gene transcription alterations measured by qPCR after exposure to difenoconazole (0, 0.25, 0.5, and 1 mg/L) at 120 h (mean ± SEM, *n* = 3). The data are expressed as means of 3 replicate samples (30 larvae/replicate) after logarithmic conversion based 2 in the heat map. The red (data > 0) indicates upregulation of gene expression, while the green (data < 0) indicates downregulation of gene expression.

**Figure 7:**
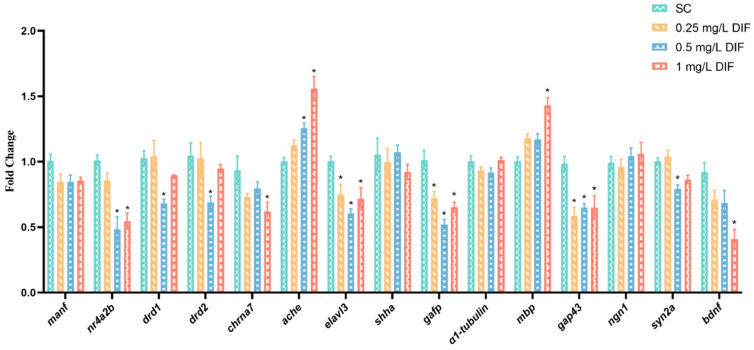
Gene transcription analysis of zebrafish after exposure to difenoconazole for 120 h (mean ± SEM, *n* = 3 replicates). * *p <* 0.05 indicates significant differences between exposure groups and the control group.

The mRNA expression of genes related to nervous system development were assessed in difenoconazole-exposed groups of zebrafish larvae at 120 hpf (Figure 6 and Figure 7), including *manf* (mesencephalic astrocyte derived neurotrophic factor), *nr4a2b* (nuclear receptor subfamily 4 group a member 2b), *drd1* (dopamine receptor D1), *drd2* (dopamine receptor D2), *chrna7* (cholinergic receptor nicotinic alpha 7 subunit), *ache*, *elavl3* (ELAV like neuron-specific RNA binding protein 3), *shha* (sonic hedgehog a), *gfap* (glial fibrillary acidic protein), *α1−tubulin*, *mbp* (myelin basic protein), *gap43* (growth associated protein 43), *ngn1* (neurogenin 1), *syn2a* (synapsin IIa), and *bdnf* (brain-derived neurotrophic factor). The transcription levels of *α1−tubulin*, *ngn1*, *manf*, and *shha* were not altered significantly relative to the control group. The notable downregulation of the expression of *bdnf* (1.58-fold) and *chrna7* (0.51-fold) were found in the 1 mg/L exposure group (*p* < 0.05) When exposed to 0.5 mg/L difenoconazole, *syn2a*, *drd1*, and *drd2* were significantly downregulation (0.26-, 0.51-, and 0.52-fold, respectively) (*p* < 0.05). At the 0.5 and 1 mg/L treatment groups, *nr4a2b* was noticeably downregulated relative to the control group by 1.09- and 0.85-fold, respectively (*p* < 0.05). The expression levels of *elavl3* (0.34-, 0.65-, and 0.40-fold), *gap43* (0.68-, 0.52-, and 0.52-fold), and *gfap* (0.40-, 0.94-, and 0.55-fold) were downregulated in the 0.25 mg/L, 0.5 mg/L, and 1 mg/L difenoconazole-exposed groups, respectively (*p* < 0.05). Conversely, *mbp* was remarkably upregulated in the 1 mg/L difenoconazole group (*p* < 0.05). The transcription of *ache* strikingly increased in the 0.5 mg/L (1.26-fold) and 1 mg/L (1.55-fold) difenoconazole-treated groups (*p* < 0.05).

## 4. Discussion

In the current study, we used zebrafish to assess the effects of difenoconazole exposure on embryonic development. The heart is the first organ formed in a developing embryo [34]; moreover, Mu et al. [35] reported that zebrafish embryos exposed to 2.0 mg/L difenoconazole showed decreased heart rate, hatching regression, and teratogenic effects (yolk sac edema, pericardial edema, and spine deformation). In another study, zebrafish embryos exposed to 5 μM difenoconazole showed decreased hatching rate, increased mortality, decreased heart rate, and severe teratogenicity (spinal curvature, yolk sac malformation, yolk sac edema, pericardial edema, and hyperemia) [36]. Similar to previous studies, we found that zebrafish heart rate and body length showed a reduction following acute exposure to difenoconazole. Difenoconazole markedly enhanced the malformation of zebrafish larvae, with pericardial edema being the most frequent type of malformation. These results suggest difenoconazole exposure could result in zebrafish developmental abnormalities. Similar abnormalities have been reported in other exposure experiments of triazole fungicides, such as prothioconazole [37] and cyproconazole [38]. It was noted the swimming behavior of zebrafish larvae changed as the concentration of difenoconazole increased, indicating difenoconazole could induce neurologic abnormality in fish. It has been reported that exposure to several triazole fungicides (propiconazole, triadimefon, and prothioconazole) could induce developmental toxicity. These studies mainly focused on the cardiotoxicity and preproductive toxicity [39,40]. Difenoconazole-induced neurotoxic effects in fish—and its potential molecular mechanisms—still need to be further clarified.

Behavior is a representation of neural activity and can be used to evaluate the neurotoxicity of drugs and substances in the environment [41,42]. Spontaneous movement is the initial motor activity generated by the growing brain network [43]. In our study, spontaneous movement in the 1 mg/L difenoconazole-treated group was significantly increased comparing to the control group at 24 hpf. Teng et al. [44] reported a similar result showing spontaneous movement of zebrafish embryos at 24 hpf were increased when exposed to difenoconazole. Similar results have also been obtained in other triazole fungicides such as penconazole [45] and propiconazole [46]. Typically, embryos at 24 hpf show 3–5 bursts per minute, accompanied by a period of dormancy [47]. Baraban et al. [48] classified the increased number of spontaneous movements at 24 hpf as epileptic movements. Swimming behavior is a more complicated activity that develops later in development and is particularly sensitive to environmental contaminants [49,50]. After 5 days of exposure to difenoconazole, the average swimming speed and total swimming distance of zebrafish larvae were considerably reduced. Previous research has demonstrated the hypoactivity of zebrafish results in impaired nervous system development, which may increase their susceptibility to predation during early development [51]. When zebrafish larvae were exposed to other triazole fungicides, the activity of the larvae was also altered. After treatment with propiconazole for 5 days, the average activity and movement distance of zebrafish larvae were significantly reduced [42]. Swimming speed was also dramatically decreased after exposure to 2 mg/L penconazole [38]. A series of behavioral function tests in this experiment can reflect the adverse effects of difenoconazole on the zebrafish nervous system.

To elucidate the underlying mechanisms of developmental neurotoxicity induced by difenoconazole, we further detected the DA content in zebrafish larvae. We discovered DA levels tend to decline in a concentration-dependent manner in difenoconazole exposure groups. DA is a neurotransmitter that plays a crucial role in the regulation of neural development, movement, and mood in zebrafish larvae [52]. In zebrafish, a decrease in DA content may cause neurotoxicity, presenting as a declined state of activity and unresponsiveness [53]. Azole pesticides have been demonstrated to inhibit calcium influx, leading to a reduction in DA neurotransmission [54]. Since gene expression is a potential marker for rapid screening of development neurotoxicity [55], we detected the mRNA expression levels of DA receptor genes and found that *drd1* and *drd2* were significantly downregulated in this study. Dopamine receptors D1 and D2 are the most abundantly expressed receptors in the brain, and furthermore, are in charge of movement regulation [56]. A previous study has shown that zebrafish larvae exhibit hypoactivity after being dosed with D1 and D2 receptor antagonists [57]. Kung et al. [58] reported that after exposure to deltamethrin, the expression of *drd1a* and *drd2a* were reduced, altering the locomotor activity of zebrafish larvae in a synergistic manner. A similar downward trend was observed at the transcription level of *nr4a2b*. The knockdown of *nr4a2* could significantly reduce the formation and differentiation of dopaminergic neurons in zebrafish [59]. Therefore, difenoconazole may alter the swimming behavior of zebrafish larvae by decreasing DA levels and interfering with dopaminergic signaling in this experiment.

Difenoconazole-induced development neurotoxicity might potentially be mediated through the cholinergic system. In this experiment, the content of ACh was decreased and the activity of AChE was increased in the difenoconazole exposure groups. ACh is the main neurotransmitter in the cholinergic system, which exists in the neuromuscular junction and the central nervous system [60]. AChE is a crucial enzyme that regulates the metabolism of ACh and is frequently employed as a biomarker to assess the toxicity of organophosphate and carbamate pesticides [61,62]. An increase in AChE activity could over-hydrolyze ACh, which would probably lead to inadequate muscular contraction and behavioral responses in zebrafish larvae [63]. Gene transcription of *ache* and *chrna7* changed significantly after exposure to difenoconazole. The α-7 nicotinic acetylcholine receptor (encoded by the gene *chrna7*) responds to the binding of ACh, while the level of AChE enzymatic activity is determined by the expression level of the gene *ache* [64], which was consistent with the changes of the content of ACh and the activity of AChE. We speculate that difenoconazole might cause hypoactivity of zebrafish larvae via interfering with the operation of the cholinergic system.

Genes and transcription factors regulate the process of brain development in zebrafish, and aberrant gene expression could result in neural development abnormalities [55]. We further analyzed the alterations of gene expression associated with neural development in this study. The neural-specific RNA-binding protein Huc, encoded by *elavl3*, is considered as a biomarker of early neuron development in zebrafish [65]. *bdnf* was induced during the period of primary nervous system formation [66], and was involved in promoting neurogenesis, increasing synaptic plasticity, and maintaining neuronal cell survival [67]. In this study, the expression levels of *elavl3* and *bdnf* were downregulated significantly, indicating that difenoconazole has detrimental effects on neuronal development and differentiation. *gfap* mainly encodes the intermediate filament protein present in astrocytes, where it is involved in and maintains the structure of their cytoskeleton [68]. Changes in *gfap* expression may modify astrocyte morphology, which affects brain structures and normal neuronal function [69]. As a biomarker of myelination, the gene *mbp* is expressed in oligodendrocytes of the central nervous system and in Schwann cells of the peripheral nervous system [70]. Upregulation of *mbp* mRNA expression indicated an enhanced protective function of microglia on oligodendrocyte maturation [71]. The increase of *mbp* expression level in this experiment may reflect the compensation of the nervous system for the damaging effect of difenoconazole. *gap 43* encodes an axonal membrane protein that is essential for neurite growth, synapse formation, and neuroplasticity during neurogenesis [72]. It is also used as a marker for re-induction of axonal growth during regeneration after nerve injury [73]. The decreased transcription levels of *gap43* might imply that difenoconazole could lead to impaired neuronal development and nerve regeneration. Correspondingly, a significant decrease in *syn2a* expression was also observed. *syn2a* is a gene that regulates the formation of synaptic structures, and its abnormal expression affects synapse formation, neurotransmitter release, and ultimately, contributes to behavioral disorders [54]. The changes of *syn2a* gene expression level may lead to neurobehavioral disorders in zebrafish larvae [74]. Similarly, exposure to penconazole, another triazole fungicide, significantly altered the transcript levels of multiple genes including *gfap*, *bdnf*, *elavl3*, *mbp*, and *gap43*, resulting in behavioral changes in zebrafish larvae [38]. In taking these results together, our findings support the hypothesis that difenoconazole exposure could trigger altered expression of genes related to nervous system development, thereby leading to the neurodevelopmental toxic effects of difenoconazole in zebrafish embryos.

## 5. Conclusions

In summary, our results demonstrated that difenoconazole exposure could induce severe developmental and neurotoxic effects in zebrafish embryos. Our findings indicated the significant changes in the transmission of the cholinergic and dopaminergic systems, as well as the transcription of key genes related to neurodevelopment might contribute to developmental neurotoxicity induced by difenoconazole. Our study provides useful information for risk assessment of the toxic effects of difenoconazole and has theoretical significance and application value for guiding the scientific use and environmental safety evaluation of phenotrimethoxazole. The neural response mechanisms behind the behavioral abnormalities are complicated. Furthermore, there is a need to conduct in-depth assessment studies on pesticide toxicity and elucidate its toxic mechanism of action to provide reference for food safety, pesticide-use safety, and human health risk assessment and standard setting.

## Figures and Tables

**Table 1:** Sequences of primers for the genes tested.

Gene Name	Sequence of the Primer (5′–3′)	Accession Number
*β−actin*	Forward: ATGGATGAGGAAATCGCTGCC Reverse: CTCCCTGATGTCTGGGTCGTC	NM_181601.5
*mbp*	Forward: AATCAGCAGGTTCTTCGGAGGAGA Reverse: AAGAAATGCACGACAGGGTTGACG	AY860977
*α1−tubulin*	Forward: AATCACCAATGCTTGCTTCGAGCC Reverse: TTCACGTCTTTGGGTACCACGTCA	NM_194388
*ngn1*	Forward: AAGCAGGGCAAGTCAAGAGA Reverse: ACGTCGGTTTGCAAGTATCC	AF024535
*syn2a*	Forward: GTGACCATGCCAGCATTTC Reverse: TGGTTCTCCACTTTCACCTT	NM_001002597.2
*elavl3*	Forward: AGACAAGATCACAGGCCAGAGCTT Reverse: TGGTCTGCAGTTTGAGACCGTTGA	NM_131449
*gap43*	Forward: TGCTGCATCAGAAGAACTAA Reverse: CCTCCGGTTTGATTCCATC	NM_131341
*bdnf*	Forward: ATAGTAACGAACAGGATGG Reverse: GCTCAGTCATGGGAGTCC	NM_131595.2
*manf*	Forward: AGATGGAGAGTGTGAAGTCTGTGTG Reverse: CAATTGAGTCGCTGTCAAAACTTG	NM_001076629
*gafp*	Forward: GGATGCAGCCAATCGTAAT Reverse: TTCCAGGTCACAGGTCAG	NM_131373
*chrna7*	Forward: TCAGTATTTTGCCACCACCA Reverse: CTTTGTCTTCGCCAGGTCTC	NM_201219.2
*ache*	Forward: CCCTCCAGTGGGTACAAGAA Reverse: GGGCCTCATCAAAGGTAACA	NM_131846.2
*nr4a2b*	Forward: GAAGACGGCGAAATCGATGC Reverse: CTGGCGGTTCTGACAACTTCC	NM_001002406.1
*drd1*	Forward: TGGTTCCTTTCTGCAACCCA Reverse: AGTGATGAGTTCGCCCAACC	NM_001135976.2
*drd2*	Forward: TCCACAAAATCAGGAAAAGCGT Reverse: CAGCCAATGTAAACCGGCAA	XM_005157501.4
*shha*	Forward: GCAAGATAACGCGCAATTCGGAGA Reverse: TGCATCTCTGTGTCATGAGCCTGT	NM_131063.3

**Table 2:** Developmental parameters in zebrafish larvae after exposing to difenoconazole (0, 0.25, 0.5, and 1 mg/L) until 120 h. * indicates significance between control and treatment groups (*p* < 0.05).

	Control (0 mg/L)	0.25 mg/L	0.5 mg/L	1 mg/L
Survival rate (%)	90.00 ± 3.85	85.56 ± 2.22	84.44 ± 2.22	83.33 ± 1.92
Hatching rate (%)	94.99 ± 0.74	92.40 ± 0.56	93.40 ± 1.58	91.79 ± 1.50
Heart rate (times/60 s)	176.60 ± 3.40	174.30 ± 2.56	169.20 ± 3.08	164.20 ± 2.74 *
Malformation rate (%)	1.00 ± 0.86	2.09 ± 1.05	5.55 ± 1.94	18.28 ± 2.51 *
Body length (mm)	4.03 ± 0.03	3.96 ± 0.03	3.92 ± 0.03	3.91 ± 0.03 *

## Data Availability

No new data were created or analyzed in this study. Data sharing is not applicable to this article.
